# Three-year weight change and risk of all-cause, cardiovascular, and cancer mortality among Iranian adults: over a decade of follow-up in the Tehran Lipid and Glucose Study

**DOI:** 10.1186/s12889-022-14126-4

**Published:** 2022-09-16

**Authors:** Niloofar Deravi, Seyyed Saeed Moazzeni, Mitra Hasheminia, Reyhane Hizomi Arani, Fereidoun Azizi, Farzad Hadaegh

**Affiliations:** 1 Prevention of Metabolic Disorders Research Center, Research Institute for Endocrine Sciences, Shahid Beheshti University of Medical Sciences, No. 24, Parvaneh Street, Velenjak Tehran, Iran; 2 Student Research Committee, School of Medicine, Shahid Beheshti University of Medical Sciences, Tehran, Iran; 3 Endocrine Research Center, Research Institute for Endocrine Sciences, Shahid Beheshti University of Medical Sciences, Tehran, Iran

**Keywords:** Body weight changes, Mortality, Cause of death, Cardiovascular diseases, Cancer

## Abstract

**Background:**

We investigated the impact of weight change on mortality in a population-based cohort setting.

**Methods:**

We conducted two weight measurements for 5436 participants aged ≥ 30 years with an approximate 3-year interval. Based on their weight change, we categorized participants to: > 5% weight loss, 3–5% weight loss, stable weight (± < 3%), 3–5% weight gain, > 5% weight gain. We followed participants for mortality annually up to March 20th 2018. We applied the multivariable Cox proportional hazard models to estimate hazard ratios (HRs) and 95% confidence intervals (CIs) of weight change categories for all-cause, cardiovascular (CV), and cancer mortality, considering stable weight as reference. The Cox models was adjusted for age, sex, educational level, body mass index, smoking status, hypertension, hypercholesterolemia, diabetes, and cardiovascular disease (CVD) at baseline.

**Results:**

During a median follow-up of 14.4 years, 629 deaths (247 CV and 126 cancer deaths) have occurred. Over 5% weight loss and gain were associated with increased risk of all-cause mortality in multivariable analysis with HRs of 1.47 [95% CI: 1.17–1.85] and 1.27 [1.02–1.57], respectively; however, a 3–5% loss or gain did not alter the risk of all-cause mortality significantly. These significant risks for wight change > 5% were not modified by the presence of diabetes, obesity, and smoking status; however, the unfavorable impact of weight change on mortality events was more prominent in those older than > 65 years (P-value for interaction: 0.042). After excluding those with history of CVD, diabetes, and cancer during the weight measurements period, these associations significantly attenuated (HR: 1.29 [0.89–1.87] for > 5% weight loss and 1.12 [0.84–1.50] for > 5% weight gain). Additionally, a > 5% weight loss was also associated with about 60% higher risk for CV mortality (HR: 1.62 [1.15–2.28]), and a 3–5% weight loss was associated with about 95% higher risk of cancer mortality (HR: 1.95 [1.13–3.38]).

**Conclusions:**

Our findings showed a U-shaped association across weight change categories for all-cause mortality risk with over 5% weight gain and loss causing higher risk. Moreover, weight loss can have adverse impact on CV and cancer mortality events.

**Supplementary Information:**

The online version contains supplementary material available at 10.1186/s12889-022-14126-4.

## Introduction

Obesity is a major public health concern. In 2016, the prevalence of obesity was more than 20% among men and more than 30% among women in most of the countries of the Middle East and North Africa (MENA) region; however, the worldwide prevalence of obesity was 11.6% for men and 15.7% for women [1]. Almost all countries of the MENA region are in nutritional transition from a traditional to a modern diet that is heavy in processed foods and fast. Therefore, their burden of disease has already shifted from communicable to non-communicable diseases (NCD). In 2013, the mean energy intake in most countries of MENA region was reported higher than the global average [1]. Moreover, a progressive increase of the fat contribution in the diet was found in most countries of this region [2]. Furthermore, air pollution is of crucial significance in the MENA, since it has some of the highest levels of ambient air pollution worldwide. A potential role of ambient air pollution in the development of obesity has also been previously proposed [3].

According to the data from the STEPwise approach to surveillance (STEPS) survey, the prevalence of overweight/obesity among Iranian adults aged 20–65 years increased from 57.8% in 2007 to 62.8% in 2016 [4]. Moreover, according to STEPs 2016, the prevalence of overweight/obesity among Iranian adults aged 65–69 years and ≥70 years were 69.7 and 55.5%, respectively [5].

As a major risk factor, high body mass index (BMI) attributed to 18.8% of deaths and 12.9% of disability-adjusted life years (DALYs) of NCDs in 2019 in Iran [6]. A J- or U-shaped relation between BMI and mortality was already established that both underweight and obesity categories were at higher mortality risk [7, 8]. Only a single measurement of BMI/weight was included in several previous cohort studies [7–9], which ignores the dynamic aspect of body weight over time. Therefore, the evaluation of long/short term consequences of weight change during certain life periods is also of high importance.

A meta-analysis of 25 cohort studies reported that among individuals aged 40–65 years, weight loss and weight gain were associated with almost 45% and 7% increased all-cause mortality risk, respectively; the corresponding values were 50% and 21% for cardiovascular (CV) mortality risk, respectively [10]. Similarly, a recent meta-analysis of 30 prospective studies reported that compared with stable weight, both weight loss and weight gain were associated with 59% and 10% increased risk of all-cause mortality, respectively, among older adults[11]. It should be noted that in both of these meta-analyses, significant heterogeneities were reported among included studies (I^2^ ranged from 41%-89%). Ethnic/Racial differences have also been evidenced in body composition [12], obesity status [13], as well as weight management behavior [14]. Consequently, the association between weight change and longevity could also vary across ethnic/racial groups [15]. To the best of our knowledge, no study has evaluated the impact of weight change on all-cause, CV, and cancer mortality risk in the MENA region. We aimed to investigate the impact of 3-year weight change on mortality rates using a large-scale, population-based cohort of Iranian adults with more than a decade of follow-up.

## Materials and methods

### Study design and study population

The Tehran Lipid and Glucose Study (TLGS) is a prospective cohort study conducted on a representative sample of residents of Tehran, the capital of the Islamic Republic of Iran.

The TLGS was designed to investigate the prevalence and incidence of NCDs and their risk factors among Iranian population [16]. Tehran was comprised of 20 urban districts at the start of the TLGS. The district no. 13 was chosen for sample selection. The rationales for selecting district 13 were: (1) high stability of the population residing in district 13 compared to other districts of Tehran, and (2) the age distribution of the population of district 13 was similar to the age distribution of the overall Tehran population [16]. Details, measurement methods, and enrollment strategy of the TLGS have been described elsewhere [17]. Briefly, in the first phase (1999–2002), 15,005 individuals aged ≥ 3 years were enrolled in the study using a multistage stratified cluster random sampling technique, and re-examinations were conducted at approximately 3-year intervals. Another 3550 individuals were added in the second phase (2002–2005) and were followed in a triennial manner.

**Fig. 1:**
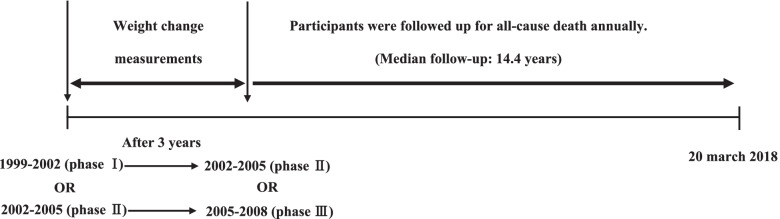
Timeline of the study design: the Tehran Lipid and Glucose Study, Iran, 1999–2018

For this study, we selected 9558 participants aged ≥ 30 years from phase 1 and 2, as the baseline population, and identified their weight change in the next phase with an interval of about 3 years. For those individuals who were enrolled at phase 1, weight change was identified in phase 2, and for participants who were enrolled at phase 2, weight change was measured in phase 3 (2005–2008). From the 9558 eligible participants, 4084 participants were excluded due to missing data on weight measurement (at baseline or next follow-up visit) or covariates at baseline. Moreover, we excluded 38 participants with no follow-up data. Finally, 5436 participants remained, who were followed up for all-cause death. Participants were censored at the date of loss to follow-up or study end (20 march 2018) (Fig. 1)**.**

We obtained written informed consent from all participants. This study was approved by the ethical committee of the Research Institute for Endocrine Sciences of Shahid Beheshti University of Medical sciences.

### Clinical and laboratory measurements

At each visit, we used interviewer-administered questionnaires to obtain demographic information, medication usage, past medical history, educational level, and smoking habits. We measured weight by a digital scale to the nearest 100 g and height in a standing position while participants had light clothing and no shoes on. Furthermore, we calculated BMI as weight in kilograms divided by the square of height in meters. Subsequent to 15 min of rest, two physician-measured blood pressures were performed on the right arm using a standard sphygmomanometer. We assessed systolic blood pressure (SBP) and diastolic blood pressure (DBP) as the mean of these two blood pressure measurements. We took morning blood samples from all participants after at least 12 h of fasting. We also performed measurements of fasting plasma glucose (FPG) and total cholesterol (TC) by standard methods, as described in detail before [16].

### Definition of terms

We defined diabetes mellitus as one of these criteria: a) FPG ≥ 7 mmol/L and b) taking any glucose-lowering drugs. Furthermore, we defined hypertension as these three criteria: SBP ≥ 140 mmHg, or DBP ≥ 90 mmHg, or using antihypertensive drugs as hypertension. Also, we defined having TC ≥ 5.18 mmol/L or using lipid-lowering drugs as hypercholesterolemia [17].

Based on smoking habits, we divided our participants into two groups: a) current smokers, b) past/never smokers. We categorized educational levels into 3 groups: 1) more than 12 years, 2) between 6–12 years, and 3) less than 6 years of academic education.

We calculated weight change as: $\frac{\mathrm{Follow}-\mathrm{up}\;\mathrm{measurement}-\mathrm{Baseline}\;\mathrm{measurement}}{\mathrm{Baseline}\;\mathrm{measurement}}\times100$. Based on 3-year weight change percentage, as recommended by Stevens et al. [18], we categorized participants into five groups: a) more than 5% weight loss; b) 3% to 5% weight loss; c) less than 3% weight change [reference group]; d) 3% to 5% weight gain; e) more than 5% weight gain.

### Outcome assessment

Details of the TLGS outcome collection have been explained previously [19]. To summarize, through an annual phone call, a trained nurse interviewed participants for any new medical events. In cases of mortality, a verbal autopsy was performed using a standard questionnaire. The questionnaire consists of time and location (in home or hospital) of death plus medical events or complications leading to death. We collected medical data for each deceased person by referring to medical record departments of service providers (outpatient or hospital). The collected data was assessed by a panel of specialists included an internist, a cardiologist, an endocrinologist, a pathologist, and an epidemiologist. The outcome committee adjudicated an underlying cause of death for each deceased participant.

### Statistical analyses

Baseline characteristics of the respondents (study participants) and non-respondents (those with missing data of main exposure/covariates or those without follow-up data) were compared. The Student’s t-test and the Chi-square test for continuous and categorical variables were used, respectively. We also illustrated baseline characteristics across weight change categories as number (%) for categorical variables and mean ± standard deviation (SD) for continuous variables.

Based on literature review[10, 11, 20], confounding factors were selected. Then, to assess the relation of weight change categories with incident all-cause, CV, and cancer mortality, we applied the multivariable Cox proportional regression analysis, and the hazard ratios (HRs) with 95% confidence intervals (CIs) were reported in two models: Model 1: adjusted for age and sex; Model 2: Model 1 + further adjusted for educational level, BMI, smoking status, hypertension, hypercholesterolemia, diabetes, and cardiovascular disease (CVD) at baseline. Multicollinearity of independent variables was checked via the variance inflation factor (VIF) statistic; given the VIF of < 4, we did not find evidence of collinearity in the model.

As a sensitivity analysis, to eliminate the effects of unintentional weight loss, participants with CVD, diabetes, and cancer at baseline or first follow-up were excluded and the association of weight change categories with all-cause mortality was reassessed.

We also checked the interactions of weight change categories with age groups (≥ 65 years versus < 65 years), sex (men versus women), BMI groups (≥ 30 kg/m^2^ versus < 30 kg/m^2^), diabetes (yes versus no), and smoking status (past/never versus current) via the log–likelihood ratio test in the multivariable model, in separate models.

Time to event is described as the time of censoring or the death occurring, whichever came first. We censored individuals in the case of leaving the district, lost to follow-up, or being alive in the study until March 20th 2018.

To assess proportionality in the Cox models, we used the Schoenfeld residual test; our proportionality assumptions were all appropriate. We employed STATA version 14 (StataCorp LP, College Station, Texas) for statistical analyses. P-values of < 0.05 were considered statistically significant.

## Results

Our study population consisted of 5436 participants (2395 men) with a mean age of 47.9 (SD: 12.1) years at baseline.

As shown in Additional file 1: Table S1, compared to non-respondents, respondents were older, less educated, had higher BMI and total cholesterol, but had lower prevalence of CVD and current smoking. Moreover, no difference was found for mortality events between groups.

Baseline and the first follow-up characteristics of the individuals across weight change categories are presented in Table 1. During the first three years of the follow-up, almost 42% of the subjects had a stable weight (-3% to + 3%). Furthermore, 27% and 9% of the participants had a weight gain or weight loss of more than 5%, respectively. Generally, in the total population, after 3 years of follow-up, BMI and FPG increased among continuous variables. Moreover, the prevalence of CVD and usage of glucose lowering, antihypertensive, and lipid-lowering drugs were increased; while SBP, DBP, total cholesterol, and current smoking were decreased.

**Table 1:** Baseline characteristics of the participants across weight change categories at the baseline and after 3-year follow-up: the Tehran Lipid and Glucose Study (TLGS), Iran, 1999–2018

Weight change categories	*Lost* > *5%*		*Lost 3% to 5%*		*Stable (*± *3%)*		*Gained 3% to 5%*		*Gained* > *5%*		*Total*	
Number of participants (Men)	533 (210)		418 (186)		2285 (1051)		731 (321)		1469 (627)		5436 (2395)	
	Baseline	Follow-up	Baseline	Follow-up	Baseline	Follow-up	Baseline	Follow-up	Baseline	Follow-up	Baseline	Follow- up
Continuous variables, Mean ± SD												
Age (year)	51.9 ± 12.4	55.3 ± 12.5	50.3 ± 12.3	53.6 ± 12.4	48.9 ± 11.9	52.1 ± 12.0	47.2 ± 11.9	50.4 ± 11.8	44.4 ± 11.4	47.9 ± 11.4	47.9 ± 12.1	51.2 ± 12.1
BMI (kg/m^2^)	29.1 ± 4.8	26.7 ± 4.5	28.5 ± 4.7	27.6 ± 4.5	27.9 ± 4.1	28.1 ± 4.2	27.3 ± 4.4	28.5 ± 4.6	26.5 ± 4.5	29.0 ± 4.9	27.6 ± 4.4	28.3 ± 4.5
SBP (mmHg)	127.6 ± 21.2	120.8 ± 21.7	125.6 ± 20.8	121.8 ± 20.3	123.9 ± 20.3	121.8 ± 19.8	120.8 ± 18.7	120.8 ± 20.7	117.3 ± 17.9	118.9 ± 19.3	122.2 ± 19.9	120.8 ± 20.0
DBP (mmHg)	80.7 ± 11.5	75.1 ± 11.4	79.6 ± 11.4	75.4 ± 10.6	79.6 ± 11.0	76.7 ± 10.7	78.5 ± 10.8	76.5 ± 10.8	77.1 ± 10.3	76.5 ± 10.5	78.9 ± 11.0	76.4 ± 10.7
FPG (mmol/L)	6.3 ± 2.5	6.4 ± 3.2	6.1 ± 2.4	6.1 ± 2.6	5.7 ± 2.0	5.8 ± 2.0	5.4 ± 1.6	5.5 ± 1.4	5.3 ± 1.6	5.4 ± 1.3	5.6 ± 2.0	5.7 ± 2.0
Total cholesterol (mmol/L)	5.9 ± 1.3	5.2 ± 1.1	5.7 ± 1.2	5.1 ± 1.0	5.7 ± 1.2	5.2 ± 1.1	5.6 ± 1.2	5.2 ± 1.0	5.4 ± 1.1	5.2 ± 1.1	5.6 ± 1.2	5.2 ± 1.1
Categorical variables, number (%)												
Educational level, years												
≤ 6	280(52.5)	277(52.0)	197(47.1)	199(47.6)	1054(46.1)	1021(44.7)	294(40.2)	294(40.2)	546(37.2)	531(36.1)	2371(43.6)	2322(42.7)
6–12	202(37.9)	206(38.6)	187(44.7)	180(43.1)	973(42.6)	999(43.7)	360(49.2)	353(48.3)	737(50.2)	741(50.4)	2459(45.2)	2479(45.6)
> 12	51(9.6)	50(9.4)	34(8.1)	39(9.3)	258(11.3)	265(11.6)	77(10.5)	84(11.5)	186(12.7)	197(13.4)	606(11.1)	635(11.7)
Current smoking	50(9.4)	49(9.2)	65(15.6)	58(13.9)	269(11.8)	272(11.9)	102(14.0)	92(12.6)	209(14.2)	194(13.2)	695(12.8)	665(12.2)
History of CVD, yes	46(8.6)	73(13.7)	26(6.2)	37(8.9)	134(5.9)	202(8.8)	43(5.9)	57(7.8)	64(4.4)	102(6.9)	313(5.8)	471(8.7)
Diabetes mellitus, yes	119(22.3)	125(23.5)	75(17.9)	81(19.4)	281(12.3)	337(14.7)	47(6.4)	73(10.0)	81(5.5)	121(8.2)	603(11.1)	737(13.6)
Hypertension, yes	203(38.1)	157(29.5)	134(32.1)	122(29.2)	691(30.2)	618(27.0)	165(22.6)	177(24.2)	295(20.1)	319(21.7)	1488(27.4)	1393(25.6)
Hypercholesterolemia, yes	380(71.3)	266(49.9)	288(68.9)	221(52.9)	1461(63.9)	1159(50.7)	457(62.5)	367(50.2)	780(53.1)	687(46.8)	3366(61.9)	2700(49.7)
Glucose-lowering drugs use, yes	61(11.4)	71(13.3)	37(8.9)	54(12.9)	134(5.9)	204(8.9)	28(3.8)	37(5.1)	45(3.1)	72(4.9)	305(5.6)	438(8.1)
Anti-hypertensive drugs use, yes	79(14.8)	87(16.3)	55(13.2)	58(13.9)	244(10.7)	275(12.0)	59(8.1)	88(12.0)	122(8.3)	132(9.0)	559(10.3)	640(11.8)
Lipid-lowering drugs use, yes	34(6.4)	32(6.0)	25(6.0)	36(8.6)	113(4.9)	130(5.7)	33(4.5)	30(4.1)	40(2.7)	60(4.1)	245(4.5)	288(5.3)
All-cause mortality events (number)	108		58		260		68		135		629	
CV mortality events (number)	52		24		104		19		48		247	
Cancer mortality events (number)	17		18		46		17		28		126	

*SD* Standard deviation, *BMI* Body mass index, *SBP* Systolic blood pressure, *DBP* Diastolic blood pressure, *FPG* Fasting plasma glucose, *CVD* Cardiovascular disease

For participants enrolled at phase I(1999–2001) of TLGS, phase II (2001–2005) was considered as the follow-up for the calculation weight change

For participants enrolled at phase II (2001–2005) of TLGS, phase III(2005–2008) was considered as the follow-up for the calculation weight change

**Fig. 2:**
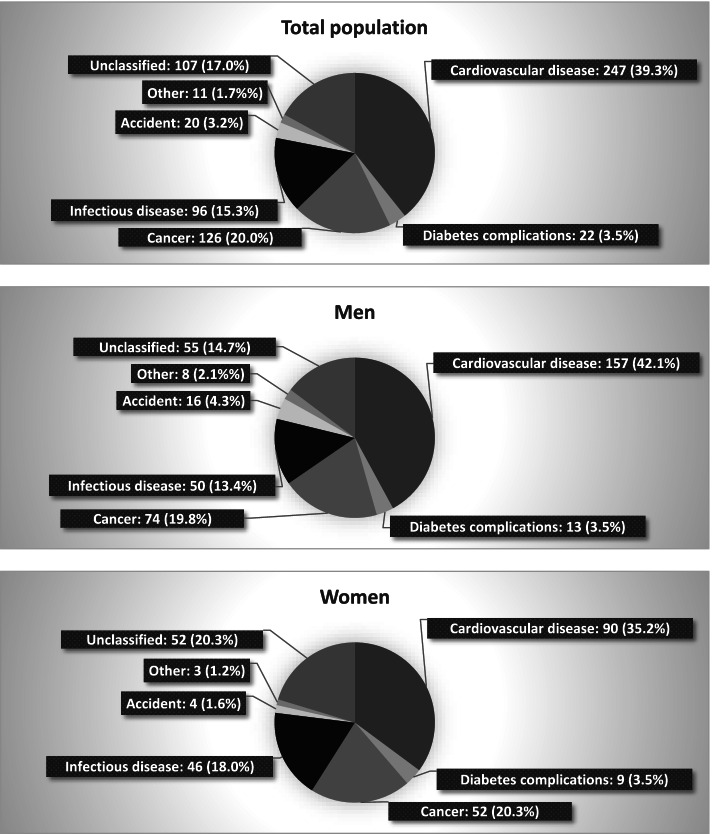
The distribution of causes of death in total population, men, and women

During a median follow-up of 14.4 years of [interquartile range: 12.7–15.5], 629 deaths (373 among men) have been recorded. The distribution of different causes of death is shown in Fig. 2**.** Underlying causes of mortality in the total population were CV (*n* = 247), cancer (*n* = 126), infectious diseases (*n* = 96), accidents (*n* = 20), diabetes complications (*n* = 22), and others (*n* = 11). Moreover, 107 cases of death had not a classified cause.

The multivariable HRs and 95% CIs of the association between weight change categories and all-cause mortality risk are shown in Table 2. Compared to subjects with stable weight, those who lost and gained more than 5% of weight had age- and sex-adjusted HRs of 1.61 [95% CI: 1.29–2.02] and 1.22 [0.99–1.50; P-value: 0.066] for the risk of all-cause mortality, respectively; the corresponding risks in model 2 were 1.47 [1.17 -1.85] and 1.27 [1.02–1.57], respectively. Importantly, male sex, older age, having less than 6 years of education, current smoking, history of CVD, diabetes, and hypertension were significantly associated with increased risk of all-cause mortality in model 2 (data not shown). After exclusion of those with history of CVD, diabetes, and cancer at baseline or first follow-up, 4294 participants remained, with a total number of 321 cases of death during follow-up. Generally, no significant association was remained; however, a suggestive (but not significant) 30% higher risk was found for the weight loss of over 5%. (Additional file 2: Table S2)**.**

**Table 2:** Multivariable hazard ratios (HR) and 95% confidence intervals (CI) of the association between weight change categories and all-cause mortality: the Tehran Lipid and Glucose Study, Iran, 1999–2018

	**Model 1**		**Model 2**	
	**HR (95% CI)**	**P-value**	**HR (95% CI)**	**P-value**
**Weight change categories**				
** Lost > 5%**	**1.61 (1.29–2.02)**	**< 0.001**	**1.47 (1.17 -1.85)**	**0.001**
** Lost 3% to 5%**	1.04 (0.78–1.39)	0.775	1.04 (0.78–1.38)	0.811
** Stable (± 3%)**	Reference		Reference	
** Gained 3% to 5%**	0.90 (0.69–1.18)	0.448	1.00 (0.76–1.30)	0.978
** Gained > 5%**	1.22 (0.99–1.50)	0.066	**1.27 (1.02–1.57)**	**0.029**

Model 1: adjusted for age and sex. Model 2: Model 1 + further adjusted for body mass index, educational level, smoking status, hypertension, hypercholesterolemia, diabetes mellitus, and history of cardiovascular disease at baseline

**Fig. 3:**
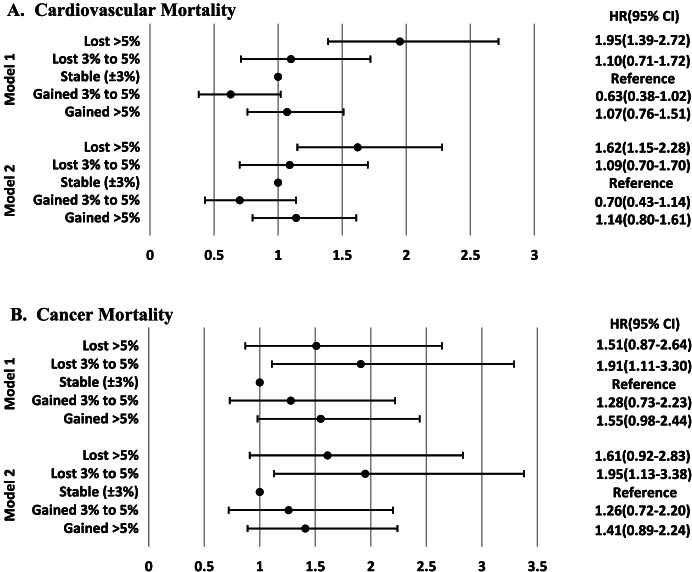
Multivariable hazard ratios (HR) and 95% confidence intervals (CI) for the association of weight change categories with cardiovascular mortality (**A**) and cancer mortality (**B**). Model 1: adjusted for age and sex; Model 2: further adjusted for body mass index, educational level, Smoking status, hypertension, hypercholesterolemia, diabetes mellitus, and history of CVD at baseline

Fig. 3 shows the associations of weight change categories with CV and cancer mortality events. As shown in Fig. 3-A, for CV mortality, a > 5% weight loss was significantly associated with increased risk (HR: 1.62 [1.15–2.28]). Moreover, after excluding those with prevalent CVD at baseline (313 participants), the results did not change (Additional file 3: Fig. S1**)**. In our data analysis, a 3–5% weight loss was also associated with an increased risk for cancer mortality events by a HR of 1.95 [1.13–3.38] (Fig. 3-B).

**Fig. 4:**
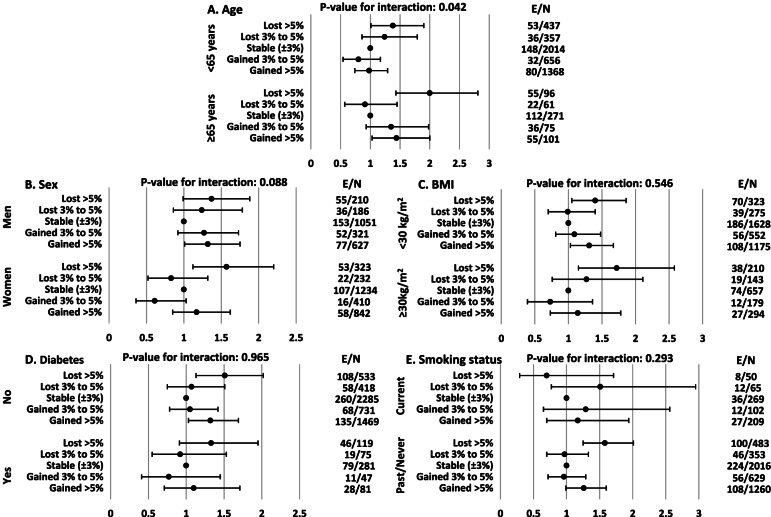
Multivariable hazard ratios and 95% confidence intervals, stratified by age (**A**), sex (**B**), BMI (**C**), Diabetes (**D**), and smoking status (**E**). E/N: Number of event/ Number of participants; BMI: body mass index; Multivariable hazard ratios were adjusted for age, sex, BMI, educational level, SMK, hypertension, hypercholesterolemia, and history of CVD at baseline; considering that age in A, sex in B, BMI in C, Diabetes in D, and smoking status in E were excluded from the models

Multivariable HRs and 95% CIs of the subgroup analysis are presented in Fig. 4. Considering age stratification, the interaction between age groups (≤ 65 years versus > 65 years) and weight change categories was significant with a P-value of 0.042. Weight loss of > 5% increased the risk of all-cause mortality in both age groups with a greater effect size for those aged > 65 years (HR: 2.01 versus 1.38); however, weight gain had a significant impact only among the older population (HR: 1.44 [1.03–2.00]). The interaction of weight change categories with sex had also a P-value of 0.088; weight gain caused more prominent adverse effects among men; however, weight loss of over 5% increased the risk of mortality in both sexes. Moreover, although the interactions of weight change categories with BMI categories, diabetes, and smoking status were not significant, in line with the total population, generally, gaining and losing weight of more than 5% was found to be significantly associated with higher risk of all-cause mortality among non-obese (BMI < 30 kg/m^2^), non-diabetes participants, as well as never/past smokers.

## Discussion

In this study, with more than a decade of follow-up, after adjustment for a large set of covariates, compared to the stable weight, participants with a > 5% weight loss or weight gain had significantly higher risk of all-cause mortality. These significant risks were not modified by the presence of diabetes, obesity, and smoking status; however, the unfavorable impact of weight change on mortality events was more prominent in the older population. Moreover, compared to women, men were more sensitive to the impact of weight gain on mortality events. Additionally, a >5% weight loss was also associated with about 60% higher risk for CV mortality, and a 3-5% weight loss was associated with about 95% higher risk of cancer mortality.

Comparing the findings of this study with other studies is not simple due to the differences in the mean age and other baseline characteristics of the participants, the sample size, considerable variations in the definitions of weight change categories, and level of adjustments for confounders. In the current study, we found a U-shaped association between weight change and all-cause mortality events. A large-scale Korean cohort reported a reverse J-shaped association between 4-year weight change and all-cause mortality risk, regardless of BMI categories [21]. A similar association was also recently reported in a multi-ethnic cohort in the United States among native Hawaiians, Japanese Americans, African Americans, whites, and Latinos [22]. A large population-based cohort study on middle-aged and elderly Chinese demonstrated a U-shaped association between weight change and all-cause/CV mortality risk, with both moderate-to-large weight gain and loss conferring excess risk compared to the nadir risk for stable weight [23]. Among the UK population in the European Prospective Investigation into Cancer in Norfolk cohort, it was shown that compared to the stable weight, weight loss was associated with higher mortality; however, findings for weight gain were inconclusive [24].

The significantly higher risk of weight loss for all-cause mortality was also addressed in two important meta-analyses. Firstly, in a meta-analysis of 25 prospective studies, it is reported that weight loss was related to 45% increased risk of all-cause mortality in middle and older age [10]. Another one showed that weight loss increased all-cause mortality risk by 59% in older adults ≥ 65 years [11]. Likely, in our data analysis, the impact of > 5% weight loss was more pronounced among older participants than the younger age group (100% versus 38% increased risk for mortality, respectively). Weight loss can be related to loss in fat and also muscle or lean body mass, particularly relevant among an aging population (sarcopenia). Since the recovery of muscle mass loss is difficult, weight loss in older adults is regarded problematic [25–27]. While on the contrary, individuals who maintain body weight in later life could be more likely to maintain muscle and bone mass compared to those losing weight [28, 29]. Undiagnosed pre-existing diseases could also be a plausible explanation for the observed increase in mortality risk among those who lost weight, especially for unintentional weight loss; however, in the current study, only 46 (7.3% of total mortality) deaths have occurred during the first two years of follow up; hence, this issue might not play a significant role in our population.

Additionally, in our study, individuals with a weight gain of > 5% were also at higher risk of mortality; the association was more prominent in older adults. This is in line with findings from the two previous meta-analyses conducted among adults aged 40–65 years [10] and specifically among older adults aged 65 years or above [11]. Since excess adiposity is proved to increase the mortality risk [7, 30], weight gain is assumed to heighten mortality risk. Weight gain is also known to increase the risk of CVD, which may also heighten mortality risk [31]. Importantly, we found that gaining weight was associated with more unfavorable impact among men, and its association was demonstrated even as little as more than 3% weight gain. It was suggested that weight gain was more attributable to the accumulation of visceral adipose tissue among men that significantly associated with poor outcomes [32].

Regarding cause specific mortality, in this study, a weight loss of > 5% showed a significant increased risk of CV mortality in the multivariable model; however, such association was not observed for weight gain. The meta-analysis of 25 studies [10], as well as two recent Chinese studies [33, 34], reported an association of both weight loss and weight gain with increased risk of CV mortality. Additionally, a 3 to 5% weight loss was associated with an increased risk of cancer mortality. This can be described by the fact that cancer-associated weight loss is associated with poor prognosis in advanced malignancy [35]. The study by Li et al. did not report significant risk of cancer related mortality among BMI change groups in overall population; however, a 5% decrease in BMI was associated with 14% increase in the risk of cancer-related mortality among men [36]. Another study from UK also reported that both weight gain and loss could increase the risk of cancer-related mortality by 17 and 14%, respectively [37].

This study has important strengths, including its prospective nature with long comprehensive follow-up. Furthermore, to the best of our knowledge, this study is the first to examine weight change and the risk of all-cause mortality in the MENA region. Finally, some previous studies related to the effect of weight change were based on self-reported questionnaires, which may have a recall information bias; however, based on the physical examination, our study used actual measurements of anthropometric indices and confounding factors.

We also acknowledge several limitations. First, due to the lack of available data, it was unknown whether weight change was unintentional or intentional. Intentional weight loss for health improvement is proved to be associated with lower mortality [38], particularly for obese individuals; therefore the exclusion of those intentionally losing weight could affect the findings of this study. Importantly, when we excluded those with prevalent comorbidity at the baseline, which potentially might have unintentional weight loss, those with weight loss more than 5% still had about 30% higher risk of mortality events that did not reach to the significant level. Second, data on some potential residual confounders, including silent comorbidities, previous weight fluctuations, socioeconomic status (excluding educational level), diet, and daily energy intake were not available; the issue might affect our results. Moreover, due to using different tools for physical activity level assessment in phases I (Lipid Research Clinic questionnaire) and II (Modifiable Activity Questionnaire), physical activity and its change were not considered as covariates; however, in national studies, it was shown that more than 21% of Iran population were physically inactive in 2011 [39]. Third, certain subgroup analyses could still be underpowered due to the small number of participants in certain strata, which may have led to insignificant associations in some categories. Therefore, the subgroup analyses findings should be extrapolated with caution. Forth, about 40% of eligible population at the baseline did not enter the data analysis; however, the mortality rate did not differ between respondents versus not respondents. This issue might indicate that the impact of older age, lower education, higher BMI and total cholesterol among respondents for mortality events was attenuated by the lower prevalence of CVD and current smoking. So, the selection bias might not apply to our data analysis. Fifth, we could not investigate the weight change in different age stages or through a longer period due to the limited sample size. Finally, the present study only included Tehranian participants and is not a national representative; hence, results cannot be generalized to the other ethnicities or rural populations.

## Conclusion

In this large-scale population-based cohort study of Iranian adults, during more than 14 years of follow-up, 3-year weight change demonstrated a U-shaped association with all-cause mortality risk; both weight gain and weight loss of > 5% were associated with increased all-cause mortality risk. It was also found that weight loss of over 5% and 3–5% was significantly associated with CV and cancer mortality events, respectively.

## Supplementary Information

**Additional file 1:** **Table S1.** Baseline characteristics of the respondents and non-respondents: the Tehran Lipid and Glucose Study, Iran, 1999-2018.

**Additional file 2:** **Table S2.** Multivariable hazard ratios (HR) and 95% confidence intervals (CI) of association between weight change categories and all-cause mortality among those without cardiovascular disease, diabetes, and cancer at baseline or first follow-up: the Tehran Lipid and Glucose Study, Iran, 1999-2018.

**Additional file 3:** **Figure S1.** Multivariable hazard ratios (HR) and 95% confidence intervals (CI) of association between weight change categories and cardiovascular (CV) mortality among those without history of CVD at baseline. Model 1: adjusted for age and sex; Model 2: further adjusted for Body mass index, educational level, Smoking status, hypertension, hypercholesterolemia, diabetes mellitus, and history of CVD at baseline.

## Data Availability

The datasets used and/or analyzed during the current study are available from the corresponding author on reasonable request.

## Abbreviations

BMI: Body mass index
DALYs: Disability-adjusted life years
NCDs: Non-communicable diseases
CVD: Cardiovascular disease
MENA: Middle east and north Africa
TLGS: Tehran lipid and glucose study
SBP: Systolic blood pressure
DBP: Diastolic blood pressure
FPG: Fasting plasma glucose
TC: Total cholesterol
SD: Standard deviation
HR: Hazard ratios
CIs: Confidence intervals
CVD: Cardiovascular disease
VIF: Variance inflation factor
