# Supplementary material for: Three-year weight change and risk of all-cause, cardiovascular, and cancer mortality among Iranian adults: over a decade of follow-up in the Tehran Lipid and Glucose Study
Source: BMC Public Health. 2022 Sep 16;22:1762. doi: 10.1186/s12889-022-14126-4 (PMC9482273; doi:10.1186/s12889-022-14126-4)
Supplement: Supplementary file 1 — Additional file 1: Table S1. Baseline characteristics of the respondents and non-respondents: the Tehran Lipid and Glucose Study, Iran, 1999-2018. [file 12889_2022_14126_MOESM1_ESM.docx]

| Table S1. Baseline characteristics of the respondents and non-respondents: the Tehran Lipid and Glucose Study, Iran, 1999-2018. | | | |
| --- | --- | --- | --- |
|  | **Respondents** | **Non-respondents** | **P-value** |
| Number of participants | **5436** | **4122** |  |
| Continuous variables, Mean ± SD | | | |
| Age (year) | 47.9 ± 12.1 | 47.1 ± 13.2 | **0.006** |
| BMI (kg/m^2^) | 27.6 ± 4.4 | 27.3 ± 4.8 | **0.006** |
| SBP (mmHg) | 122.2 ± 19.9 | 121.8 ± 20.7 | 0.355 |
| DBP (mmHg) | 78.9 ± 11.0 | 78.5 ± 11.2 | 0.148 |
| FPG (mmol/L) | 5.6 ± 2.0 | 5.7 ± 2.1 | 0.862 |
| Total cholesterol (mmol/L) | 5.6 ± 1.2 | 5.5 ± 1.2 | **0.003** |
| Categorical variables, number (%) | | | |
| Men | 2395 (44.1%) | 1937 (47.0%) | **0.004** |
| Educational level, years |  |  | **0.003** |
| - ≤6 | 2371 (43.6%) | 1669 (40.6%) |  |
| - 6-12 | 2459 (45.2%) | 1910 (46.5%) |  |
| - >12 | 606 (11.1%) | 527 (12.8%) |  |
| Current smoking, yes | 695 (12.8%) | 764 (19.4%) | **<0.001** |
| History of CVD, yes | 313 (6.9%) | 290 (8.6%) | **0.005** |
| Glucose-lowering drugs use, yes | 305 (5.6%) | 231 (5.6%) | 1.000 |
| Anti-hypertensive drugs use, yes | 559 (10.3%) | 388 (9.4%) | 0.167 |
| Lipid-lowering drugs use, yes | 245 (4.5%) | 164 (4.0%) | 0.221 |
| Mortality event | 629(11.6%) | 451(11.2%) | 0.601 |
| SD: Standard deviation; BMI: body mass index; SBP: systolic blood pressure; DBP: diastolic blood pressure; FPG: fasting plasma glucose; CVD: cardiovascular disease. | | | |
