# Supplementary material for: Three-year weight change and risk of all-cause, cardiovascular, and cancer mortality among Iranian adults: over a decade of follow-up in the Tehran Lipid and Glucose Study
Source: BMC Public Health. 2022 Sep 16;22:1762. doi: 10.1186/s12889-022-14126-4 (PMC9482273; doi:10.1186/s12889-022-14126-4)
Supplement: Supplementary file 2 — Additional file 2: Table S2. Multivariable hazard ratios (HR) and 95% confidence intervals (CI) of association between weight change categories and all-cause mortality among those without cardiovascular disease, diabetes, and cancer at baseline or first follow-up: the Tehran Lipid and Glucose Study, Iran, 1999-2018. [file 12889_2022_14126_MOESM2_ESM.docx]

| **Table S2. Multivariable hazard ratios (HR) and 95% confidence intervals (CI) of association between weight change categories and all-cause mortality among those without cardiovascular disease, diabetes, and cancer at baseline or first follow-up: the Tehran Lipid and Glucose Study, Iran, 1999-2018.** | | | | |
| --- | --- | --- | --- | --- |
|  | **Model 1** | | **Model 2** | |
|  | **HR (95% CI)** | **P-value** | **HR (95% CI)** | **P-value** |
| **Weight change categories** |  |  |  |  |
| - **Lost > 5%** | 1.21 (0.84-1.74) | 0.317 | 1.29 (0.89 -1.87) | 0.173 |
| - **Lost 3% to 5%** | 1.03 (0.70-1.52) | 0.882 | 1.04 (0.70-1.54) | 0.855 |
| - **Stable (± 3%)** | Reference |  | Reference |  |
| - **Gained 3% to 5%** | 0.98 (0.70-1.39) | 0.919 | 1.02 (0.72-1.45) | 0.904 |
| - **Gained > 5%** | 1.18 (0.89-1.57) | 0.254 | 1.12 (0.84-1.50) | 0.434 |
| **Model 1: adjusted for age and sex. Model 2: Model 1+ further adjusted for body mass index, educational level, smoking status, hypertension, and hypercholesterolemia at baseline.** | | | | |
