# Supplementary material for: Three-year weight change and risk of all-cause, cardiovascular, and cancer mortality among Iranian adults: over a decade of follow-up in the Tehran Lipid and Glucose Study
Source: BMC Public Health. 2022 Sep 16;22:1762. doi: 10.1186/s12889-022-14126-4 (PMC9482273; doi:10.1186/s12889-022-14126-4)
Supplement: Supplementary file 3 — Additional file 3: Figure S1. Multivariable hazard ratios (HR) and 95% confidence intervals (CI) of association between weight change categories and cardiovascular (CV) mortality among those without history of CVD at baseline. Model 1: adjusted for age and sex; Model 2: further adjusted for Body mass index, educational level, Smoking status, hypertension, hypercholesterolemia, diabetes mellitus, and history of CVD at baseline. [file 12889_2022_14126_MOESM3_ESM.docx]

**Figure S1. Multivariable hazard ratios (HR) and 95% confidence intervals (CI) of association between weight change categories and cardiovascular (CV) mortality among those without history of CVD at baseline.**

Model 1: adjusted for age and sex; Model 2: further adjusted for Body mass index, educational level, Smoking status, hypertension, hypercholesterolemia, and diabetes mellitus.
